# Constitutive Activation of Natural Killer Cells in Primary Biliary Cholangitis

**DOI:** 10.3389/fimmu.2019.02633

**Published:** 2019-11-15

**Authors:** Theresa J. Hydes, Matthew D. Blunt, Jennifer Naftel, Andres F. Vallejo, Grégory Seumois, Alice Wang, Pandurangan Vijayanand, Marta E. Polak, Salim I. Khakoo

**Affiliations:** ^1^Clinical and Experimental Sciences, Faculty of Medicine, University of Southampton, Southampton, United Kingdom; ^2^Department of Medicine, La Jolla Institute for Allergy and Immunology, University of California, San Diego, San Diego, CA, United States

**Keywords:** primary biliary cholangitis, natural killer cells, chemokine receptor 6 protein, human, interleukin-12, STAT4 transcription factor

## Abstract

Natural killer (NK) cells are innate immune cells that interface with the adaptive immune system to generate a pro-inflammatory immune environment. Primary Biliary Cholangitis (PBC) is a hepatic autoimmune disorder with extrahepatic associations including systemic sclerosis, Sjogren's syndrome and thyroiditis. Immunogenetic studies have identified polymorphisms of the IL-12/STAT4 pathway as being associated with PBC. As this pathway is important for NK cell function we investigated NK cells in PBC. Circulating NK cells from individuals with PBC were constitutively activated, with higher levels of CD49a and the liver-homing marker, CXCR6, compared to participants with non-autoimmune chronic liver disease and healthy controls. Stimulation with minimal amounts of IL-12 (0.005 ng/ml) led to significant upregulation of CXCR6 (*p* < 0.005), and enhanced IFNγ production (*p* < 0.02) on NK cells from PBC patients compared to individuals with non-autoimmune chronic liver disease, indicating dysregulation of the IL-12/STAT4 axis. In RNAseq studies, resting NK cells from PBC patients had a constitutively activated transcriptional profile and upregulation of genes associated with IL-12/STAT4 signaling and metabolic reprogramming. Consistent with these findings, resting NK cells from PBC patients expressed higher levels of pSTAT4 compared to control groups (*p* < 0.001 vs. healthy controls and *p* < 0.05 vs. liver disease controls). In conclusion NK cells in PBC are sensitive to minute quantities of IL-12 and have a “primed” phenotype. We therefore propose that peripheral priming of NK cells to express tissue-homing markers may contribute to the pathophysiology of PBC.

## Introduction

Primary Biliary Cholangitis (PBC) affects 1 in 1,000 women over 40 and is common indication for liver transplantation (1). PBC predominantly affects the liver, but is associated with a number of other conditions including Sjogren's syndrome, scleroderma, thyroid disease, and autoimmune diabetes (2). It can thus be considered a syswtemic disease. Genome wide association studies (GWAS) have consistently shown an association of polymorphisms within the Interleukin (IL)-12/Signal transducer and activator of transcription (STAT) 4 pathway with the development of PBC (3–8). This pathway has been implicated in other autoimmune and inflammatory diseases including rheumatoid arthritis, chronic active hepatitis, psoriatic arthritis and Crohn's disease (9–11).

PBC is characterized by an elevation of Th1 cytokines, including interferon gamma (IFNγ), and also Th17 cells as mediators of inflammation (12, 13). Natural killer (NK) cells produce IFNγ and are being increasingly recognized for their role in adaptive immunity and having trained immunological memory (14, 15). Furthermore, they have been demonstrated to modulate tumor microenvironments toward inducing cytotoxic T-cell responses (16). Thus, NK cells have the potential to play an important role in autoimmune disease. NK cells have been previously implicated in the pathogenesis of PBC (17, 18). In PBC NK cells demonstrate enhanced cytotoxic functions (19), are found in increased numbers around the small bile ducts and can be induced to lyse biliary epithelial cell (BECs) following toll-like receptor stimulation (20). Consistent with a role in interfacing with the adaptive immune system, NK cells may potentiate CD4 T-cell responses in PBC (21).

Recent work has shown that NK cells may also have “adaptive” features, with one characteristic being enhanced IFNγ production (22, 23). Additionally CXCR6 marks liver-resident NK cells (24) and CD49a is present on tissue-resident NK cells in humans, including some liver-resident NK cells (23, 25–27). Both are associated with adaptive NK cells in murine studies (28, 29). Consistent with this observation, human liver-resident CD49a+ NK cells have a strong Th1 cytokine response and display adaptive features (23, 25). We recently showed that IL-12 can upregulate both CXCR6 and CD49a expression on NK cells in the peripheral blood, with comparable phenotypic features to liver-resident CD49a+ NK cells, linking NK cell peripheral activation with liver-homing (25). Based on these observations we aimed to investigate NK cell dysfunction in PBC.

## Materials and Methods

### Patients

Patients were recruited from University Hospital Southampton NHS Foundation Trust with informed consent (Table 1) and full ethical approval (Research Ethics Committee numbers 13/WA/0329 and 06/Q1701/120).

**Table 1:** Characteristics of individuals with PBC and control groups.

	**Primary biliary cholangitis** **(*n* = 36)**	**Haemochromatosis** **(*n* = 31)**	**Healthy controls** **(*n* = 9)**	***p*****-value**		
				**PBC vs.** **HFe**	**PBC vs.** **HC**	**HFe vs.** **HC**
Age at time of study, median (range)	61 (44–83)	51 (20–73)	33 (20–40)	0.002	<0.001	0.007
Men, *N* (%) Women, *N* (%)	3 (8.3) 33 (91.7)	24 (77.4) 7 (22.6)	0 (0) 9 (100)	<0.001	0.370	<0.001
Cirrhosis, *N* (%)	7 (19.4)	7 (22.6)	0 (0)	0.753	0.150	0.117
UDCA, *N* (%)	28 (80.6)	0 (0)	0 (0)	<0.001	<0.001	-
Co-existent autoimmune disorder, *N* (%)	9 (25.0)	2 (6.5)	0 (0)	0.0410	0.094	0.434

### PBMC Isolation and Cell Surface Staining

Peripheral blood mononuclear cells (PBMCs) were isolated from individuals with PBC, haemochromatosis (HFe), and healthy controls (HC) using Ficoll-Paque™ density centrifugation (GE Healthcare, Sweden). PBMCs were stained with CD3 (UCHT1, BV510, Biolegend®, London, UK), CD56 (HCD56, PE-Cy7, Biolegend®), CD49a (SR84, PE, BD Biosciences), CXCR6 (K041E5, PerCP/Cy5.5, Biolegend®), and analyzed by flow cytometry using FlowJo v.10.0 (Treestar, USA). Gates were set using fluorescence minus one controls.

### RNA Sequencing

CD49a+ and CD49a- peripheral CD3-CD56+ NK cells from PBC patients, and CD3-CD56+ NK cells from HC were sorted using a BD FACS Aria directly into TRIzol (ThermoFisher, MA). RNA was isolated using miRNeasy micro kit (Qiagen, Hilden, Germany) loaded on an automated platform (Qiacube, Qiagen). Samples were quantified as described previously (30, 31) and quality of RNA assessed by Fragment Analyzer (Advance Analytical). All samples had an RNA integrity number > 7.5. Purified total RNA (≈5 ng) was amplified following the Smart-seq2 protocol (32, 33). Briefly, mRNA was captured using poly-dT oligos and reverse-transcribed into full-length cDNA using the described template-switching oligo (32, 33). cDNA was amplified by PCR, purified using AMPure XP magnetic beads (Beckman Coulter). One nanogram of cDNA was used to prepare a standard NextEra XT sequencing library (NextEra XT DNA library prep kit and index kits; Illumina). Barcoded Illumina sequencing libraries (Nextera; Illumina) were generated utilizing an automated platform (Biomek FXP, Beckman Coulter). Both whole-transcriptome amplification and sequencing library preparations were performed in a 96-well format to reduce assay-to-assay variability. Quality control steps were included to determine total RNA quality and quantity, the optimal number of PCR preamplification cycles, and fragment library size. Samples were pooled at equimolar concentration, loaded and sequenced on the Illumina Sequencing platform, HiSeq2500 (Illumina) to obtain more than 7 million 50-bp single-end reads (HiSeq Rapid Run Cluster and SBS Kit V2; Illumina) mapping uniquely to mRNA reference. Reads were mapped to ENSEMBL (34) release 95 using kallisto (35) with bias correction, and 50 bootstrap samples. Differentially expressed genes (DEG) were found using EdgeR (36) aggregating transcripts to gene level. All models included a term to model individual variation. Main differences of PBC vs. HC were detected using a model with group effect. CD49a+ vs. CD49a- NK cells were compared using a paired design. Genes with a false discovery rate (FDR)-corrected *P*-value < 0.05 were identified as differentially expressed, resulting from a likelihood ratio test using a negative binomial generalized linear model fit. Normalization offsets were calculated using the TMM. Pre-ranked gene set enrichment analysis was performed using Fgsea R package (37) with 10,000 permutations and the Molecular Signatures Database v 6.2(MsigDB) (38). Heat maps were generated using gplots v3.01 (39) clustering the samples by spearman correlation. Pathways were created using ReactomeDB (40) and visualized using Cytoscape v3.7 using the fold changes obtained from EdgeR. The project number at Gene Expression Omnibus is PRJNA542532, https://www.ncbi.nlm.nih.gov/bioproject/542532.

### Functional Assays

NK cells were purified from PBMCs using an NK cell isolation kit (Miltenyi Biotec, Surrey) and cultured in R10 [RPMI 1640 + Glutamax (Gibco®, Life Technologies™) supplemented with 10% fetal bovine serum (Hyclone®, Thermoscientific, Northumberland, UK), penicillin, streptomycin, and glutamine (Gibco®, Life Technologies™)] and 5% human serum (HS) alone or with increasing concentrations of rhIL-12 (PeproTech, London, UK) and rhIL-15 (R and D Systems, Oxford, UK). For IFNγ secretion, PBMCs were stimulated for 12 h with IL-12, GolgiStop™ (BD Biosciences) was added (4 μl/6 ml culture medium) for the last 4 h, prior to cell surface staining as described above. Cells were then fixed and permeabilized (BD Cytofix/Cytoperm™ Plus Kit, BD Biosciences) prior to incubation with IFNγ (B27, APC, Biolegend®). pSTAT4 activation was assessed by flow cytometry of PBMCs at rest and following stimulation with IL-12 for 1 h. Cells were surface stained as above, then incubated at 37°C for 10–12 min in Cytofix solution (BD Biosciences), prior to resuspension in 1 ml Perm III Buffer (BD Biosciences) and staining with pSTAT4 (PY693, AF647, BD Biosciences).

For the CD107a degranulation assay, PBMCs were incubated overnight with 1 ng/ml rhIL-15 (R and D Systems) and then added to a 96-well round bottomed plate with either no target or 721.221 cells at a 5:1 or 10:1 effector:target ratio. Cells were co-incubated for 4 h at 37°C in the presence of anti-CD107a-AF647 (eBioscience Ltd, Hatfield, UK). GolgiStop (BD Biosciences) was added after the first hour of incubation and cells were then stained and analyzed by flow cytometry.

### Statistical Analysis

Statistical analysis was performed using Graph Pad Prism 7. Surface marker expression and intracellular staining for IFNγ and pSTAT4 are presented using the median and interquartile range. The Wilcoxon matched pairs signed rank test was used to compare data within the same patient group and the Mann Whitney *U*-test was used to compare data from different patient groups. The Kruskal-Wallis' test with Dunn's multiple comparison test was used to compare three unpaired sample groups. Regression analysis and the coefficient of determination (r^2^) was used to determine correlation between expression levels of CXCR6 and CD49a with age. Chi-squared test was used to compare categorical demographic data.

## Results

### CXCR6+ and CD49a+ NK Cells Are Found at Higher Frequencies in the Peripheral Blood in Patients With PBC Compared to Controls

Thirty-six individuals with PBC were enrolled (Table 1). Peripheral blood was also collected from 31 patients with HFe as non-autoimmune liver disease controls, and nine healthy volunteers. Individuals with PBC were more likely to be female and slightly older than the HFe population, however the liver disease groups were matched for disease stage. Median serum liver enzyme levels were as follows for individuals with PBC: alkaline phosphatase (ALP) 175 IU/l (range 64–1487), alanine transaminase (ALT) 27.5 IU/l (range 12–226) and Bilirubin 9 μmol/l (range 3–65).

The frequencies of *ex-vivo* unstimulated NK cells in the peripheral blood were not significantly different between participant groups: PBC 10.8%, HFe 11.4%, and HC 11.5% (Supplementary Figure 1A). Frequencies of CD56^bright^ NK cells were also comparable, with a non-significant trend toward a higher frequency of CD56^bright^ NK cells in the PBC group (PBC 8.9% vs. HFe 6.4% and HC 5.3%; Supplementary Figure 1B).

**Figure 1:**
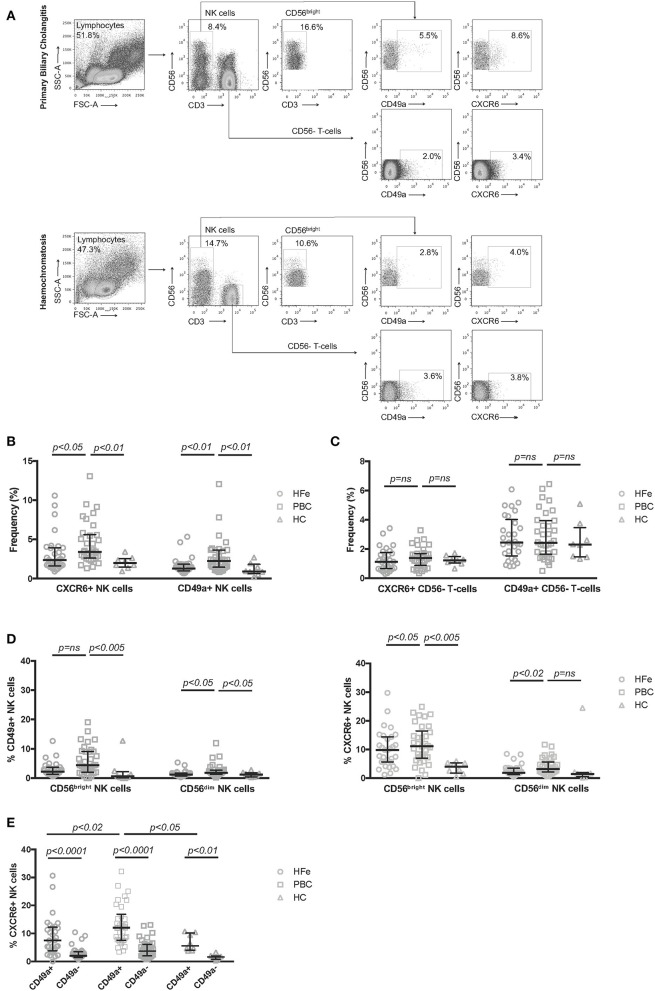
**(A)** Representative flow cytometry plots showing the gating strategy used to define CD56^bright^, CXCR6+ and CD49a+ NK cell subpopulations within PBMCs. Representative plots are shown for patients with PBC and HFe. **(B)** The frequency of CXCR6+ and CD49a+ NK cells within the peripheral CD3-CD56+ NK cell population for individuals with HFe (*n* = 31), PBC (*n* = 34), and HC (*n* = 8). **(C)** The frequency of CXCR6+ and CD49a+ CD56- T-cells within the CD56- T-cell population for individuals with HFe (*n* = 31), PBC (*n* = 34), and HC (*n* = 8). **(D)** The frequency of CD49a+ and CXCR6+ NK cells within the CD56^bright^ and CD56^dim^ NK cell populations for individuals with HFe (*n* = 31), PBC (*n* = 34), and HC (*n* = 8). **(E)** The frequency of CXCR6+ NK cells within the peripheral blood CD49a+ and CD49a- NK cell populations for individuals with HFe (*n* = 31), PBC (*n* = 34), and HC (*n* = 8). Dot plots show individual values, the median and interquartile range. Patient groups were compared using the Kruskal-Wallis test with Dunn's multiple comparison test. The Wilcoxon matched pairs test was used to compare CD49a+ and CD49a- populations within the same patient group.

The frequency of NK cells expressing CXCR6 was significantly higher in PBC patients compared to HFe (3.4 vs. 2.4%, *p* < 0.05) and HC (3.4 vs. 2.0%, *p* < 0.01; Figures 1A,B). There was also increased expression of CD49a on NK cells from PBC patients compared to HFe (2.2 vs. 1.3%, *p* < 0.01) and HC (2.2 vs. 0.9%, *p* < 0.01; Figures 1A,B). These increases were not associated with Ursodeoxycholic acid (UDCA) therapy (Supplementary Figures 2A,B) and were specific to NK cells, as there was no increase in the frequencies CD3+CD56- T-cells expressing CXCR6 or CD49a in PBC patients (Figure 1C). Examination of all participants revealed there was no association between either gender or age with frequencies of CXCR6+ and CD49a+ NK cells, suggesting that the higher frequency of tissue-resident NK cells observed in the blood in PBC patients, compared to the chronic liver disease control group, is not a result of their demographic differences (Supplementary Figure 3). In both liver disease groups, CXCR6 and CD49a were found more frequently on CD56^bright^ and CD56^dim^ NK cells as compared to HC. In PBC however there was a higher frequency of CXCR6 on CD56^dim^ NK cells, the cytotoxic subpopulation (3.2 vs. 1.9%, *p* < 0.05), and of CD49a on both the CD56^bright^ (4.4 vs. 2.2%, *p* < 0.05) and CD56^dim^ (1.8 vs. 1.2%, *p* < 0.02) NK cells, as compared to HFe controls (Figure 1D). Thus, overall there is increased expression of markers associated with tissue-residency on resting peripheral blood NK cells in PBC.

Liver-resident CXCR6+ and CD49a+ NK cells are known to be phenotypically and functionally distinct (25). To investigate the relationship between CXCR6+ and CD49a+ NK cells in the peripheral blood we examined the expression of CXCR6 on circulating CD49a+ and CD49a- NK cells *ex vivo*. We found that the CD49a+ NK cell population contained higher frequencies of CXCR6+ cells than the CD49a- population, for individuals with HFe, PBC, and HC (Figure 1E). These frequencies were higher within the CD49a+ NK cell subset for patients with PBC vs. HFe (12.0 vs. 7.5%, *p* < 0.02) and vs. HC (12.0 vs. 5.6%, *p* < 0.05) (Figure 1E).

### Circulating CD49a+ NK Cells in PBC Are Constitutively Activated

**Figure 2:**
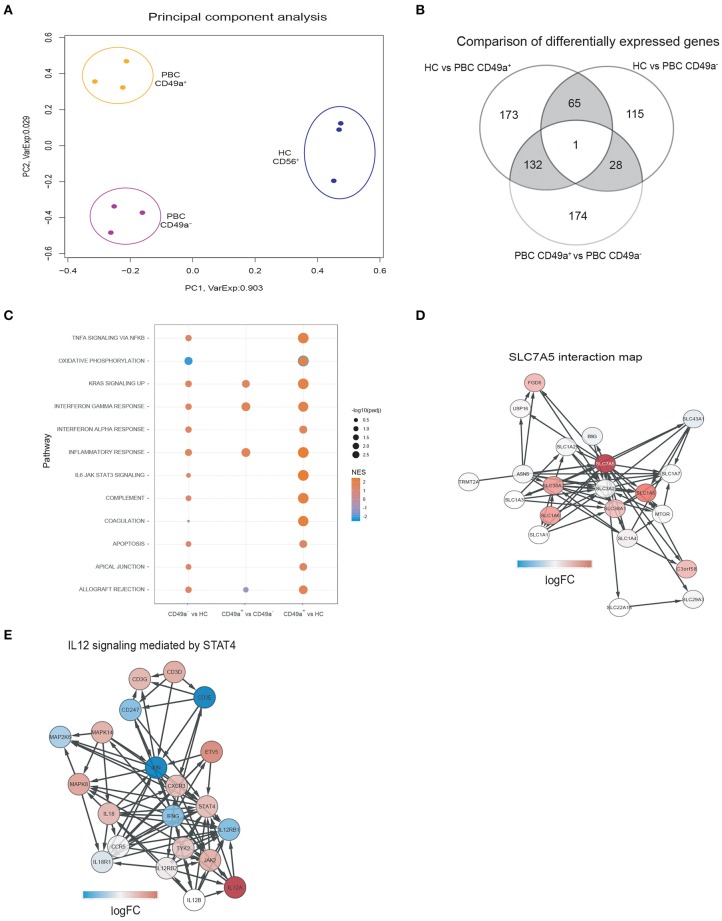
**(A)** Principal Component Analysis of gene expression profiles segregates samples in three clusters for each condition. Each dot represents the gene expression profile of a sample. The colors indicate the different patient groups: HC (blue), PBC CD49a+ (orange), and PBC CD49a- (purple). The axis labels indicate the percentages of explained variance corresponding to the represented principal component. **(B)** Comparison of differentially expressed genes (DEG) between HC and PBC CD49a+ (group comparison), HC and PBC CD49a- (group comparison), and PBC CD49a+ vs. CD49a- (paired test). DEG were found using EdgeR with a false discovery rate (FDR) less than 0.05. **(C)** Gene-set enrichment analysis (GSEA) was performed against the hallmark set (Broad institute) using each one of the three group comparisons. **(D)** SLC7A5 interactions maps, with the red color representing the logFC in the CD49a+ vs. HC comparison for the SLC7A5 interaction. The network was created using String: *p* < 0.05. **(E)** Activation of the IL-12/STAT4 pathway in PBC CD49a+ cells in comparison with HC, color represents the logFC of each gene in the comparison (red indicates upregulation, and blue downregulation). The signaling pathway was created from ReactomeDB: *p* = 0.01.

As PBC is an inflammatory autoimmune disorder and our previous work had shown that intrahepatic NK cells expressing CD49a are associated with higher levels of IFNγ than CXCR6 (25), we further characterized the CD49a+ NK cell population by performing RNA sequencing on paired sorted peripheral blood CD49a+ and CD49a- NK cells from three patients with PBC, and peripheral blood derived NK cells from three HC. Of the three individuals with PBC all were female, aged 48, 56, and 56 and one had cirrhosis. Principal Component Analysis revealed that both subsets of NK cells from PBC patients had a distinct transcriptional profile compared to NK cells from HC, and that CD49a+ NK cells from PBC patients had a divergent gene expression profile from CD49a- NK cells (Figure 2A).

Differential expression gene (DEG) analysis revealed 371 genes with an absolute log2 (fold change) >2 and FDR < 0.05, from which 210 were upregulated in CD49a+ NK cells from PBC patients compared to HC (Supplementary Table 1). In a homologous comparison with CD49a- cells, 209 DEG (164 upregulated) were found. In addition, comparing CD49a+ and CD49a- cells from paired donors yielded 335 DEG (138 upregulated). Figure 2B shows a summary of all three comparisons. A total of 66 DEG were related with PBC independent of CD49a expression, constituting a common PBC signature. Upregulated genes included *LTBP1, NECTIN2, TNFRSF10D, NKTR, TLR7, IFIT1, IFIT2, IFIT3, ERAP2*, and *MCOLN2*. In addition, 305 DEGs were exclusively detected on HC vs. PBC CD49a+ NK cells, and 132 were detected when comparing CD49a+ vs. PBC CD49a- NK cells.

Analysis between CD49a+ and CD49a- NK cells in PBC patients identified 335 DEG (FDR < 0.05). Gene ontology analysis indicated that CD49a+ NK cells had a gene signature associated with immune cell activation, including modules for the inflammatory response (FDR: E-4), immune response (FDR: E-10), major histocompatibility complex protein complex assembly (FDR: E-3), adaptive immune system (FDR: E-3), and phagocytosis (FDR: E-8). Activation-associated markers that were significantly upregulated in CD49a+ NK cells included *CD68, LYZ, NFAM1, DOK3, CD300E, TREM1, IFNGR2, CD40, CXCL16, TNFRS10D*, and *SLC7A7*. This implies that CD49a+ NK cells represent an activated and primed NK cell subset in PBC.

In comparison to HC, CD49a+ NK cells from PBC patients represent an activated phenotype characterized by enrichment of the inflammatory response (normalized enrichment score [NES]: 2.3), signaling through IL-6/STAT3 (NES: 2.3), oxidative phosphorylation (NES: −2.4), TNFα signaling (NES: 2.0), and KRAS signaling (NES: 2.0) (Figure 2C). Whereas, PBC CD49a- and CD49a+ NK cells had lower levels of genes for oxidative phosphorylation (NES: −2.1), consistent with metabolic reprogramming of NK cells. Furthermore, network analysis revealed upregulation of a family of amino acid transporters related with SLC7A5 (Figure 2D) and upregulation of BCAT1, a target of c-Myc, in PBC CD49a+ cells (logFC: 4.7). c-Myc is essential for IL-2/IL-12 induced metabolic reprogramming in NK cells and is associated with upregulation of SLC7A5 (41). We therefore analyzed cytokine signaling pathways. A total of 12 genes were upregulated (*p* < 0.05) in CD49a+ NK cells from PBC patients compared to NK cells from HC including key genes associated with cytokine signaling (*IRF8, STAT1, SOCS1, IL7R, IL12A*, and *NFKB1*). Consistent with the STAT4 pathway being dysregulated in PBC, components of the IL-12/STAT4 pathway were upregulated in CD49a+ and CD49a- NK cells compared to NK cells from HC. *STAT4, JAK2, CD3D, MAPK8, CXCR3*, and *IL12A* were significantly enriched in PBC derived CD49a+ NK cells compared to HC (Figure 2E). Furthermore, the transcription factor ZBTB32, which is associated with adaptive NK cell responses following murine cytomegalovirus infection, was also upregulated in CD49a+ compared to CD49a- NK cells from PBC patients (*p* < 0.001, FDR < 0.01, Supplementary Table 1) (42, 43).

### Low Dose IL-12 Preferentially Stimulates NK Cells From Individuals With PBC

**Figure 3:**
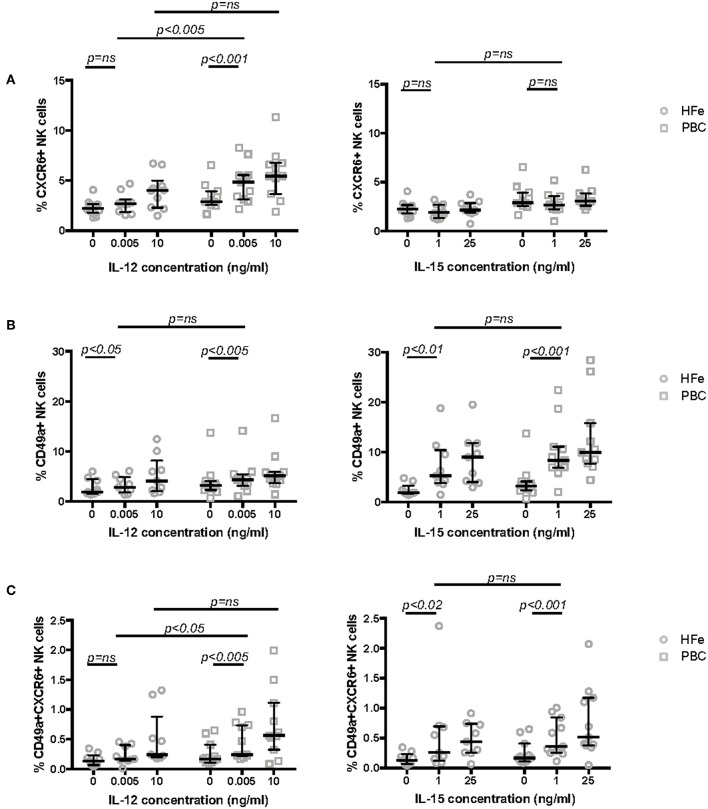
**(A)** The frequency of CXCR6+ NK cells within the peripheral blood NK cell population of non-cirrhotic individuals with HFe (*n* = 10) and PBC (*n* = 11) following stimulation of purified NK cells for 12 h in media only and with increasing concentrations of IL-12 and IL-15. **(B)** The frequency of CD49a+ NK cells within the peripheral blood NK cell population of non-cirrhotic individuals with HFe (*n* = 9) and PBC (*n* = 11) following stimulation of purified NK cells for 12 h in media only and with increasing concentrations of IL-12 and IL-15. **(C)** The frequency of CD49a+CXCR6+ NK cells within the peripheral blood NK cell population of non-cirrhotic individuals with HFe (*n* = 9) and PBC (*n* = 11) following stimulation of purified NK cells for 12 h in media only and with increasing concentrations of IL-12 and IL-15. Dot plots show individual values, the median and interquartile range. The Mann Whitney *U*-test compares patient groups and the Wilcoxon matched pairs test compares conditions within the same patient group.

To investigate whether the altered phenotype of NK cells in PBC may be related to changes in the cytokine signaling pathway, we studied the expression of CXCR6 and CD49a following cytokine activation in short term culture. We cultured purified NK cells from non-cirrhotic individuals with PBC and HFe in increasing concentrations of IL-12 (0.005, 0.5, 5.0, and 10 ng/ml) and IL-15 (1 and 25 ng/ml) for 12 h. Stimulation with 0.005 ng/ml IL-12 led to significant upregulation of CXCR6 on NK cells from individuals with PBC, from 2.9 to 4.9% (*p* < 0.001). No upregulation was observed in the HFe group (2.2 to 2.7%, *p* > 0.05). At this concentration of IL-12 NK cells from PBC patients showed significantly greater CXCR6 expression than NK cells from HFe patients (*p* < 0.005) (Figure 3A). IL-15 did not significantly upregulate CXCR6 in either patient group (Figure 3A). Conversely, we observed similar responses in CD49a expression to both IL-12 and IL-15 in the PBC and HFe groups (Figure 3B). However, stimulation with IL-12 0.005 ng/ml led to an increase in CD49a+CXCR6+ double-positive NK cells in individuals with PBC compared to those with HFe (0.24 vs. 0.17%, *p* < 0.05), and compared to culture of PBC NK cells in media alone (0.24 vs. 0.17%, *p* < 0.005) (Figure 3C). Responses to IL-15 were similar between PBC and HFe NK cells (Figures 3B,C). Thus, in PBC peripheral NK cells are sensitive to IL-12 and this preferentially upregulates the liver homing marker CXCR6.

**Figure 4:**
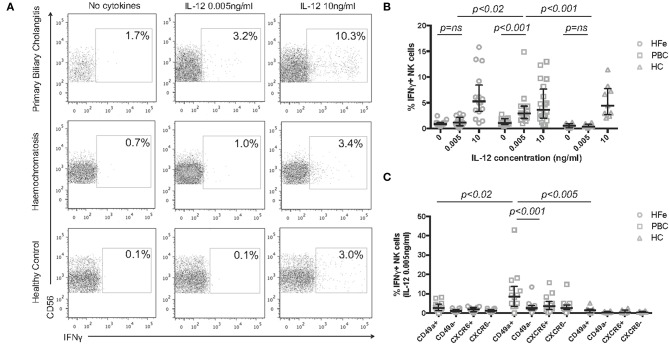
**(A)** Representative flow cytometry plots for each patient group showing the gating strategy for IFNγ+ NK cells, following gating on CD3-CD56+ NK cells, post stimulation with increasing concentrations of IL-12 for 12 h. **(B)** The frequency of IFNγ+ NK cells from the peripheral blood of individuals with HFe (*n* = 14–16), PBC (*n* = 14–20), and HC (*n* = 8–9) following stimulation with increasing concentrations of IL-12 for 12 h. **(C)** The frequency of IFNγ+ NK cells within the CD49a+, CD49a-, CXCR6+, and CXCR6- NK cell populations from the peripheral blood of individuals with HFe (*n* = 14), PBC (*n* = 14), and HC (*n* = 8) following stimulation with IL-12 0.005 ng/ml for 12 h. Dot plots show individual values, the median and interquartile range. Patient groups were compared using the Kruskal-Wallis test with Dunn's multiple comparison test. The Wilcoxon matched pairs test was used to compare IL-12 concentrations and NK cell subpopulations within the same patient group.

To determine if the IL-12 sensitivity of NK cells in PBC is associated with a functional consequence we measured IFNγ secretion. NK cells from individuals with PBC, but not from HFe patients or HC, responded to 0.005 ng/ml IL-12 with an increase in IFNγ expression from 1.1% at rest to 2.9% (*p* < 0.001) and produced greater quantities of IFNγ compared to HFe patients (2.9 vs. 1.2%, *p* < 0.02) and HC (2.9 vs. 0.4%, *p* < 0.001; Figures 4A,B). At higher concentrations of IL-12 responses were similar between the groups, suggesting that this is a hypersensitivity to IL-12 and not an increase in the potential for NK cells to secrete IFNγ (Figure 4B).

Activated and “memory” NK cells have been shown to express CD49a, which is associated with enhanced IFNγ secretion (23, 25). We therefore investigated which subsets of NK cells were associated with IFNγ release in response to low dose IL-12. CD49a+ NK cells from individuals with PBC were more readily stimulated to release IFNγ than CD49a- NK cells (median 8.6 vs. 2.5% *p* < 0.001) consistent with their activated phenotype by RNAseq. Whilst CD49a+ NK cells from HFe and HC groups also secreted IFNγ in response to low dose IL-12, this was significantly less than from PBC patients [PBC 8.6% (range 0–43.0%) vs. HFe 2.6% (range 0–8.2%), *p* < 0.02 and HC 1.4% (range 0–5.1%), *p* < 0.005] (Figure 4C). IFNγ production by the CD49a- NK cell populations was comparable between the two liver disease groups (*p* > 0.05). No significant difference in IFNγ secretion was noted between CXCR6+ and CXCR6- NK cells.

Since NK cells are thought to lyse BECs in PBC (19, 20) we also investigated the cytotoxic potential of circulating CD49a+ and CD49a- NK cells isolated from patients with PBC. For an effector:target ratio of 5:1 and 10:1 CD49a+ NK cells displayed enhanced degranulation compared to CD49a- NK cells (5:1 61.9 vs. 47.7%, 10:1 56.9 vs. 40.1%, both *p* < 0.01; Supplementary Figure 4). Therefore, the peripheral blood of patients with PBC contains a subpopulation of primed NK cells which are marked by CD49a.

### NK Cells in Individuals With PBC Have Baseline Activation of the IL-12/STAT4 Axis

**Figure 5:**
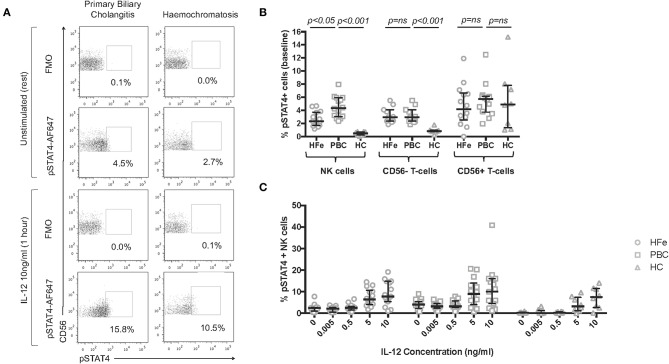
**(A)** Representative flow cytometry plots for individuals with PBC and HFe showing the gating strategy for pSTAT4+ NK cells, following gating on CD3-CD56+ NK cells, at rest and post stimulation with IL-12 10 ng/ml. **(B)** The resting frequency of pSTAT4+ NK cells [HFe (*n* = 14), PBC (*n* = 14), and HC (*n* = 8)], pSTAT4+ CD56- T-cells [HFe (*n* = 11), PBC (*n* = 11), and HC (*n* = 8)], and pSTAT4+ CD56+ T-cells [HFe (*n* = 14), PBC (*n* = 14), and HC (*n* = 8)]. **(C)** The frequency of pSTAT4+ NK cells within peripheral NK cell subpopulations from individuals with HFe (*n* = 14–15), PBC (*n* = 14–15), and HC (*n* = 8) following stimulation with increasing concentrations of IL-12 for 1 h. Dot plots shows individual values, the median and interquartile range. Patient groups were compared using the Kruskal-Wallis test with Dunn's multiple comparison test.

As IFNγ release induced by IL-12 signals via STAT4, we investigated STAT4 phosphorylation in NK cells in PBC. Individuals with both PBC and HFe had higher levels of pSTAT4+ CD56- T-cells (PBC 2.9%, HFe 2.9%, HC 0.8%; PBC vs. HFe *p* > 0.05, PBC vs. HC *p* < 0.001, HFe vs. HC *p* < 0.001) and a comparable level of CD56+ T-cells compared to HC (PBC 5.7%, HFe 4.2%, HC 4.9%, *p* > 0.05 for all). However, a higher percentage of NK cells from individuals with PBC (4.3%) were found to express pSTAT4 at rest compared to both HFe patients (2.3%) and HC (0.5%) (*p* < 0.05 and *p* < 0.001, respectively; Figures 5A,B). Thus, consistent with the RNAseq data, the STAT4 pathway is activated in PBC.

On stimulation with low doses of IL-12, the frequencies of pSTAT4+ NK cells did not increase further in either patient group until concentrations reached 5 ng/ml (Figures 5A,C). Differences between the groups were lost at this point. This indicates that the increased levels of IFNγ production observed following IL-12 0.005 ng/ml are most likely related to increased baseline levels in STAT4 activation rather than *de novo* STAT4 phosphorylation during culture, and that NK cells in PBC are constitutively activated.

## Discussion

We have identified that NK cells in PBC are activated, as determined by both phenotypic and functional analysis. Furthermore, they can be readily induced to express the liver homing marker CXCR6. The observation that CD49a can also be upregulated with low dose IL-12 in individuals with other chronic liver diseases, could be related to differences in transcriptional regulation between CXCR6 and CD49a, in that CD49a expression on NK cells appears more sensitive to short term culture with IL-12 than CXCR6. Consistent with this, previous work has shown that exogenous IL-12 has a greater effect on CD49a expression than CXCR6 expression, on NK cells in long term culture (25). However, there does appear to be a dysregulation of the IL-12 signaling pathway in PBC. We found that STAT4 was constitutively phosphorylated to greater levels in PBC than in the liver disease controls, and of functional relevance NK cells in PBC could be induced to secrete more IFNγ. Immunogenetic studies have correlated polymorphisms in the IL-12/STAT4 signaling pathway with the development of PBC (3–8). In our RNAseq analysis, genes involved in this pathway were upregulated in NK cells from PBC patients, consistent with an underlying defect of NK cells in this autoimmune disease. Interestingly STAT4 polymorphisms have been associated with other major autoimmune diseases (9–11). Thus, this could represent a common mechanism for the potentiation of autoimmune disorders.

Liaskou et al. have previously shown that T_reg_ cells from patients with PBC were highly sensitive to IL-12, but in that study IL-12 concentrations of 10 ng/ml were used (44). This is 2,000 times the concentration we used to demonstrate upregulation of CXCR6 and CD49a on NK cells from PBC patients. As the mean level of IL-12p70 found in the serum of PBC patients is around 10 pg/ml, and levels in the liver around 60 pg/ml, then the concentrations of IL-12 we tested are well within the physiological range (45). Thus in PBC, NK cells are several orders of magnitude more sensitive to IL-12 levels than T_reg_ cells. We therefore propose that the single nucleotide polymorphisms in the IL-12/STAT4 pathway are more likely to have their effects on NK cells.

The transcriptomic analysis presented here demonstrates that CD49a+ NK cells in PBC have a “primed” phenotype. Activation of NK cells is associated with metabolic reprogramming and we found upregulation of the amino acid transporter *SLC7A5* in the CD49a+ NK cell subset in PBC. This transporter is thought to be important for c-Myc mediated metabolic reprogramming of NK cells. Furthermore, there was downregulation of genes associated with oxidative phosphorylation. Thus, in PBC NK cells have features of metabolic reprogramming. Intriguingly, both metabolic reprogramming and expression of CD49a are associated with adaptive NK cells (23).

Additionally we observed upregulation of the transcription factor *ZBTB32* in CD49a+ NK cells in PBC. This has previously been described in association with cytomegalovirus seropositivity, and is thought to be essential for the proliferative burst of NK cells during viral infections (46, 47). This raises the possibility that infectious agents may prime NK cells in PBC, by stimulating secretion of IL-12, upregulating CXCR6 on NK cells, and so signaling NK cells to traffic to the liver. Such IL-12 release could be triggered by bacterial or viral infections, which could occur anywhere in the body, including urinary tract infections which have previously been associated with PBC (48). PBC has multiple extrahepatic associations and so the acquisition of a tissue-residency phenotype could be associated with recruitment of NK cells to tissues other than the liver.

Our work demonstrates that NK cells in PBC circulate with an activated phenotype and readily express markers of liver-homing and tissue-residency, suggesting that they could migrate to the liver and contribute toward a pro-inflammatory hepatic environment. Furthermore, circulating CD49a+ NK cells isolated from patients with PBC demonstrate enhanced degranulation and thus may be capable of lysing BECs. Recent work in both humans and mice has demonstrated that NK cells can be recruited from the circulation into the liver, and in humans this has been related to recruitment of CXCR6^+^Eomes^lo^ NK cells into the liver to subsequently become CXCR6^+^Eomes^hi^ (24, 49). Consistent with this, CXC Chemokine Ligand 16 (CXCL16), the chemokine that attracts CXCR6+ lymphocytes is upregulated in PBC (50). However, further studies of intrahepatic NK cells are required to determine the extent to which NK cells contribute toward hepatic inflammation. These studies may reveal if NK cells are involved in initiating inflammation, and augmenting T-cell mediated inflammation in PBC, in addition to contributing toward the lysis of BECs (51).

In conclusion our data demonstrate that NK cells in PBC circulate with a primed phenotype, suggestive of a role in the underlying pathogenesis of the disease. Investigation of NK cells in other autoimmune disorders is warranted as they represent a novel target for therapeutic intervention.

## Data Availability Statement

The datasets generated for this study can be found in the Gene Expression Omnibus, PRJNA542532, https://www.ncbi.nlm.nih.gov/bioproject/542532.

## Ethics Statement

The studies involving human participants were reviewed and approved by (1) Wales Research Ethics Committee, REC No. 13/WA/0329. (2) South Central Hampshire Research Ethics Committee, REC No. 06/Q1701/120. The patients/participants provided their written informed consent to participate in this study.

## Author Contributions

TH was responsible for patient recruitment, experimental design, acquisition of data, data analysis, and interpretation and drafting of the manuscript. MB was responsible for data acquisition, analysis and interpretation. JN was responsible for data acquisition and analysis. AV was responsible for data analysis and interpretation. GS and AW were responsible for data acquisition and analysis. PV was responsible for study and experimental design with respect to RNA sequencing and data interpretation. MP was responsible for data interpretation. SK supervised the project, was responsible for the study concept, experimental design, data interpretation and critical revision of the manuscript.

### Conflict of Interest

SK has previously received a personal speakers fee from the pharmaceutical company Dr. Falk. The remaining authors declare that the research was conducted in the absence of any commercial or financial relationships that could be construed as a potential conflict of interest.

## Abbreviations

ALT: Alanine transaminase
ALP: Alkaline phosphatase
BCAT1: Branched chain amino acid transaminase 1
BEC: Biliary epithelial cell
CD: Cluster of differentiation
CXCL16: CXC Chemokine Ligand 16
CXCR: Chemokine receptor
DEG: Differentially expressed genes
FDR: False discovery rate
GWAS: Genome wide association studies
HC: Healthy controls
HFe: Haemochromatosis
IFNγ: Interferon gamma
IL: Interleukin
NK: Natural killer
PBC: Primary biliary cholangitis
PBMCs: Peripheral blood mononuclear cells
SLC7A5: Solute carrier family 7 member 5
STAT: Signal transducer and activator of transcription
UDCA: Ursodeoxycholic Acid
ZBTB32: Zinc finger and BTB domain containing 32.
