# Supplementary material for: Constitutive Activation of Natural Killer Cells in Primary Biliary Cholangitis
Source: Front Immunol. 2019 Nov 15;10:2633. doi: 10.3389/fimmu.2019.02633 (PMC6874097; doi:10.3389/fimmu.2019.02633)
Supplement: Supplementary file 2 [file Data_Sheet_1.PDF]

SUPPLEMENTARY FIGURES

Supplementary Figure 1

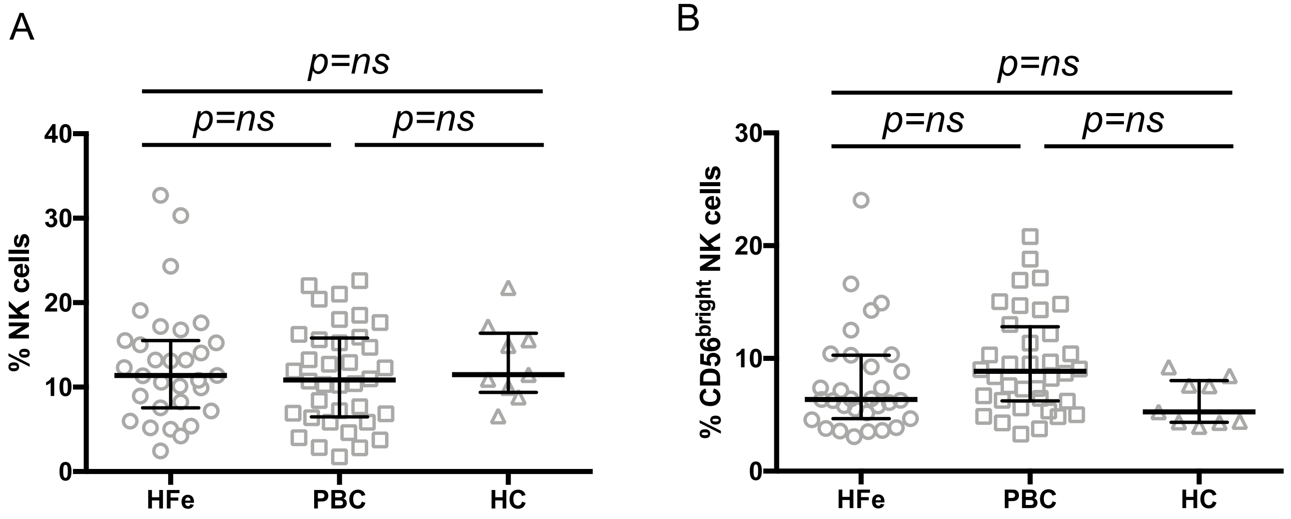

Supplementary Fig 1. A) Frequency of NK cells within the peripheral lymphocyte population for individuals with HFe (*n=31*), PBC (*n=36*) and HC (*n=9*). **B)** Frequency of CD56^{bright} NK cells within the peripheral NK cell population for individuals with HFe (*n=31*), PBC (*n=36*) and HC (*n=9*). Dot plots show individual values, the median and interquartile range. Comparisons are made using the Kruskal-Wallis test with Dunn's test for multiple comparisons.

Supplementary Figure 2

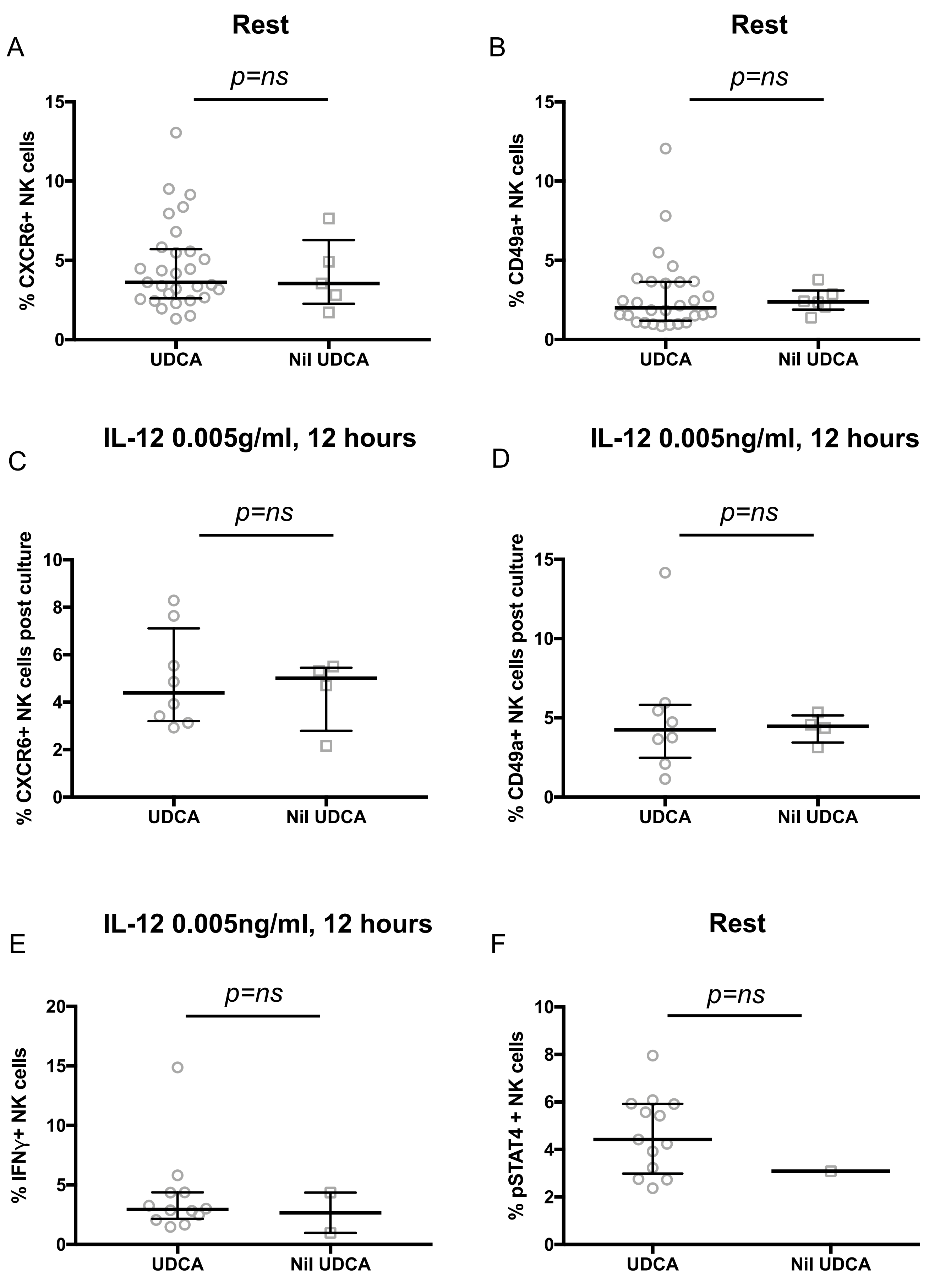

Supplementary Fig 2. A) Frequency of CXCR6+ NK cells within the peripheral NK cell population at rest in individuals with PBC according to UDCA prescription. (*n=29, n=5).* **B)** Frequency of CD49a+ NK cells within the peripheral NK cell population at rest in individuals with PBC according to UDCA prescription. (*n=29, n=6).* **C)** Frequency of CXCR6+ NK cells within the peripheral NK cell population following stimulation with IL-12 0.005ng/ml for 12 hours in individuals with PBC according to UDCA prescription. (*n=8, n=4).* **D)** Frequency of CD49a+ NK cells within the peripheral NK cell population following stimulation with IL-12 0.005ng/ml for 12 hours in individuals with PBC according to UDCA prescription. (*n=8, n=4).* **E)** Frequency of IFNγ+ NK cells within the peripheral NK cell population following stimulation with IL-12 0.005ng/ml for 12 hours in individuals with PBC according to UDCA prescription. (*n=12, n=2).* **F)** Frequency of pSTAT4+ NK cells within the peripheral NK cell population at rest in individuals with PBC according to UDCA prescription. (*n=13, n=1).* Dot plots show individual values, the median and upper interquartile range. The Mann Whitney U test was used to compare patient groups.

Supplementary Figure 3

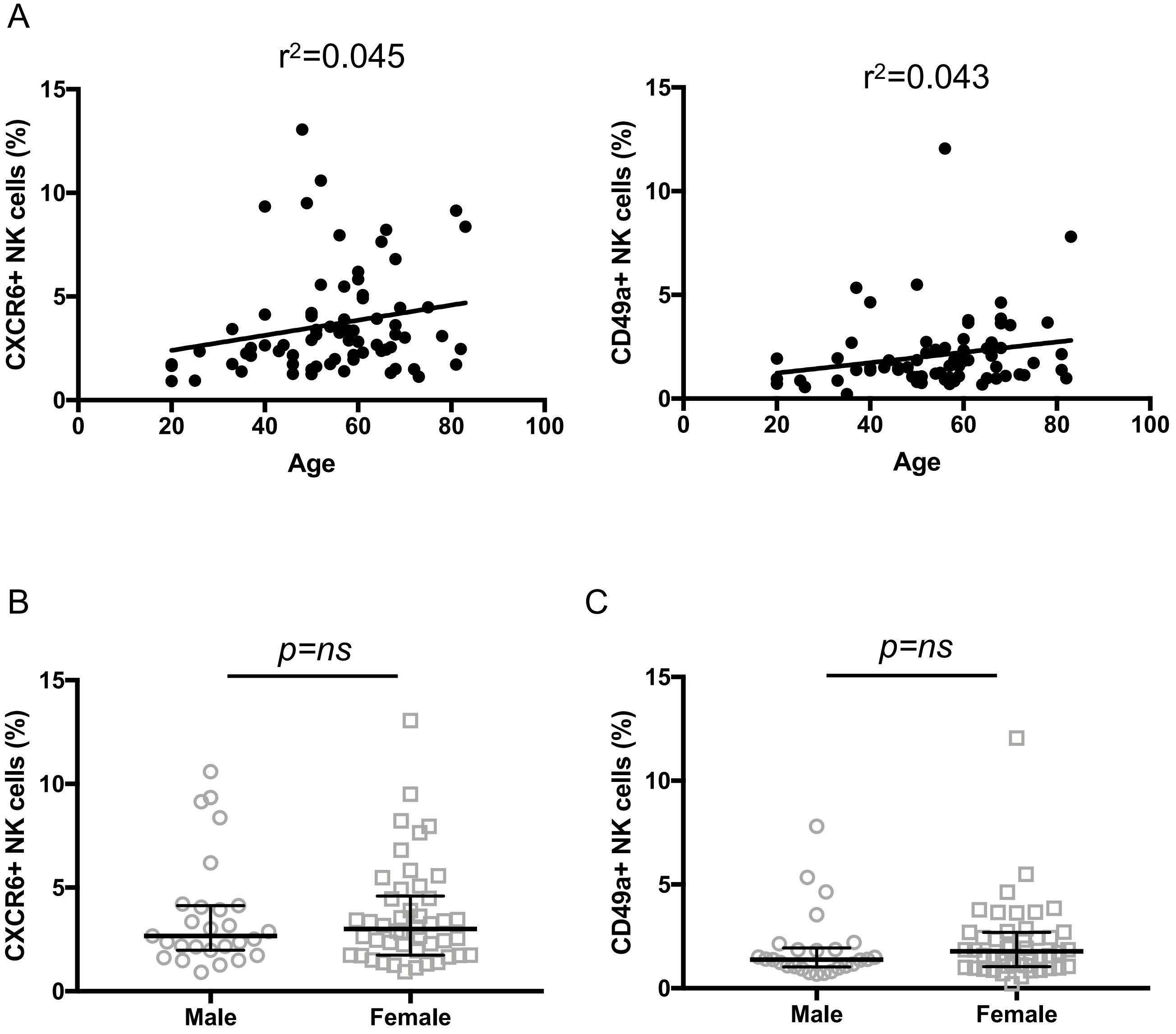

Supplementary Fig 3. A) Linear regression models of the frequencies of CXCR6+ and CD49a+ NK cells within the peripheral NK cell population at rest with age for all participants (*n=74*). The coefficient of determination (r^2) is shown. **B)** Frequency of CXCR6+ NK cells within the peripheral NK cell population at rest in all participants according to gender. (male *n=26,* female *n=46).* **C)** Frequency of CD49a+ NK cells within the peripheral NK cell population at rest in all participants according to gender. (male *n=27,* female *n=46).* Dot plots show individual values, the median and interquartile range. The Mann Whitney U test compares gender groups.

Supplementary Figure 4

A

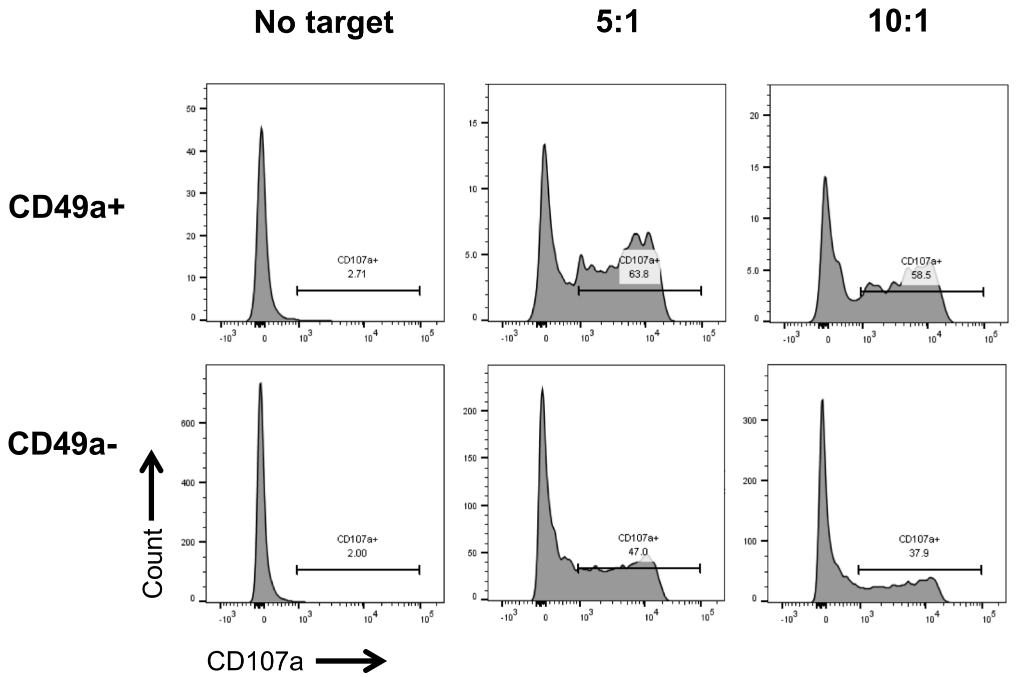

B

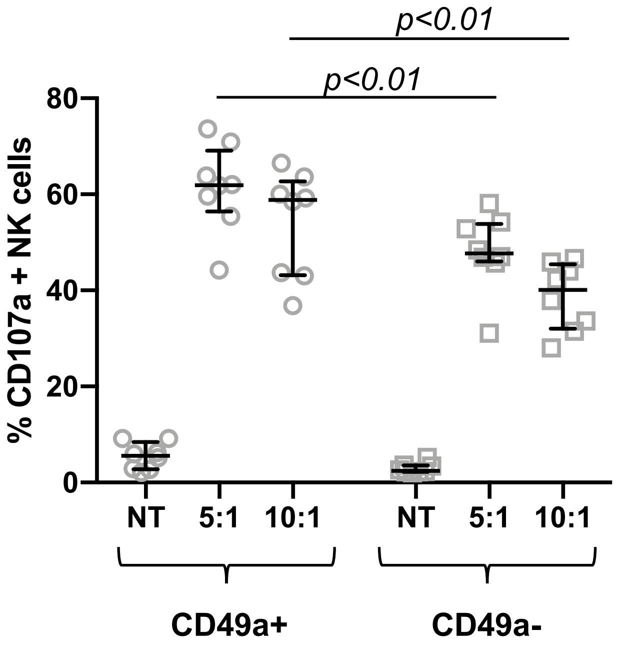

Supplementary Fig 4. A) Representative flow cytometry histograms showing the gating of CD107a+ NK cells within the CD49a+ and CD49a- NK cell populations with no target, an effector:target ratio of 5:1 and an effector:target ratio of 10:1. **B)** The frequency of CD107a+ NK cells within the CD49a+ and CD49a- NK cell populations with no target, an effector:target ratio of 5:1 and an effector:target ratio of 10:1 (*n=8*). Dot plots show individual values, the median and interquartile range. The Wilcoxon matched pairs test was used to compare CD49a+ and CD49a- populations within the same patient.
